# Psychometric properties of instruments assessing the ethics of teaching: a COSMIN-based systematic review

**DOI:** 10.3352/jeehp.2026.23.12

**Published:** 2026-05-28

**Authors:** Kesinee Chaimo, Sureeporn Thanasilp

**Affiliations:** 1 Faculty of Nursing, Chulalongkorn University, Bangkok, Thailand; 2 Asian Wisdom Care Research Unit, Faculty of Nursing, Chulalongkorn University, Bangkok, Thailand; Hallym University, Korea

**Keywords:** Cross-cultural comparison, Delivery of health care, Health occupations, Psychometrics, Workplace

## Abstract

**Purpose:**

This systematic review aimed to identify and critically evaluate instruments assessing the ethics of teaching and related moral constructs among educators, with a focus on their psychometric properties and applicability to health professions education.

**Methods:**

A systematic search was conducted in PubMed, ERIC, Scopus, and Emerald Insight databases through January 31, 2026. Only English-language studies were included. Measurement properties were evaluated using COSMIN (consensus-based standards for the selection of health measurement instruments) and COSMIN-modified GRADE (Grading of Recommendations Assessment, Development, and Evaluation) approaches.

**Results:**

Of 246 records, 6 instruments met the inclusion criteria: ESSQ (Ethical Sensitivity Scale Questionnaire), ELS (Ethical Leadership Scale), EEQ (Ethical Evaluation Questionnaire), TEPI (Teaching-Profession Ethical Principles Inventory), TCPERSS (Teachers’ Compliance with Professional Ethics in Relations with Students Scale), and MCI (Moral Competency Inventory). Psychometric properties were sufficiently reported for selected domains, primarily internal consistency and structural validity (Cronbach’s α=0.74–0.97). However, construct validity (hypothesis testing), test–retest reliability, and cross-cultural validation were inconsistently reported. The quality of evidence was moderate because of limited cross-context validation. Notably, no tools were specifically developed for health professions education. Most identified instruments focused on classroom pedagogy, potentially overlooking clinical instruction, bedside teaching, and workplace-based learning, where power dynamics and clinical pressures coexist. Developing tools that capture the “ethics of the clinical encounter” would help more accurately reflect the realities faced by health professions educators.

**Conclusion:**

Existing instruments demonstrate sufficient psychometric properties in general education but reveal critical measurement gaps for health professions education. These findings provide an empirical basis for developing context-specific instruments to improve the evaluation of ethical teaching in clinical and healthcare settings.

## Graphical abstract

[Fig f2-jeehp-23-12]

## Introduction

### Background

The ethics of teaching is a fundamental component of educational quality, fairness, and integrity. It encompasses the ethical principles and professional values that guide teachers’ behavior, decision-making, and interactions with learners. Ethical teaching is expressed both directly and indirectly through classroom instruction, clinical supervision, and academic advising, with the aim of creating safe, fair, and respectful learning environments [1,2]. In health professions education, the ethics of teaching has particular significance because educators serve as moral role models. Their conduct in both classroom and clinical settings demonstrates core values such as honesty, respect, and professionalism, which directly influence students’ professional development and patient safety [3,4].

Despite its recognized importance, assessing the ethics of teaching remains complex. Ethical behaviors involve attitudes and abstract normative principles that are difficult to operationalize and quantify [5]. Moreover, existing tools vary considerably in their conceptual frameworks and psychometric rigor, limiting their comparability and utility [6,7]. Within health professions education, these challenges are intensified. Many institutions lack instruments that have undergone comprehensive psychometric evaluation, and tools from general education often fail to capture critical dimensions relevant to healthcare, such as managing power dynamics and protecting learner rights [8,9]. To our knowledge, no prior systematic review has comprehensively synthesized and evaluated the measurement properties of instruments assessing the ethics of teaching across general and health professions education.

### Objectives

This systematic review aimed to identify and critically evaluate existing instruments designed to assess the ethics of teaching and their measurement properties, including validity, reliability, and cultural relevance. By synthesizing evidence using the COSMIN (consensus-based standards for the selection of health measurement instruments) methodology, this study sought to identify the strengths and limitations of available tools and provide a structured foundation for developing psychometrically sound and contextually appropriate instruments for diverse educational settings.

## Methods

### Ethics statement

This was a literature-based study; therefore, institutional review board approval and informed consent were not required.

### Study design

This systematic review was reported in accordance with the Preferred Reporting Items for Systematic Reviews and Meta-Analyses (PRISMA) 2020 statement [10]. The PRISMA 2020 checklist is provided as Supplement 1. This review was prospectively registered on the Open Science Framework on January 4, 2026 (https://osf.io/fxuv2). The review was conducted in strict accordance with the registered protocol, and no major deviations affecting the review objectives or eligibility criteria occurred during the review process.

### Eligibility criteria

Studies were eligible if they met the following criteria: (1) target population: teachers or educators in general or professional education; (2) focus: development, adaptation, or validation of instruments assessing the ethics of teaching or related moral constructs; (3) reporting of at least 1 psychometric property; and (4) publication in peer-reviewed English-language journals (Table 1). Exclusion criteria included studies focusing on students’ or patients’ ethics, studies lacking empirical psychometric data, and purely conceptual studies.

### Information sources

A systematic search was conducted on January 31, 2026, across PubMed, ERIC, Scopus, and Emerald Insight. No restrictions were placed on publication year to ensure maximum retrieval of relevant instruments from database inception.

### Search strategy

Search terms were developed around 4 concepts: construct of interest, target population, measurement properties, and methodology (Table 2). The full strategies and syntax for all databases are presented in Table 3.

### Selection process

**Fig. 1.**
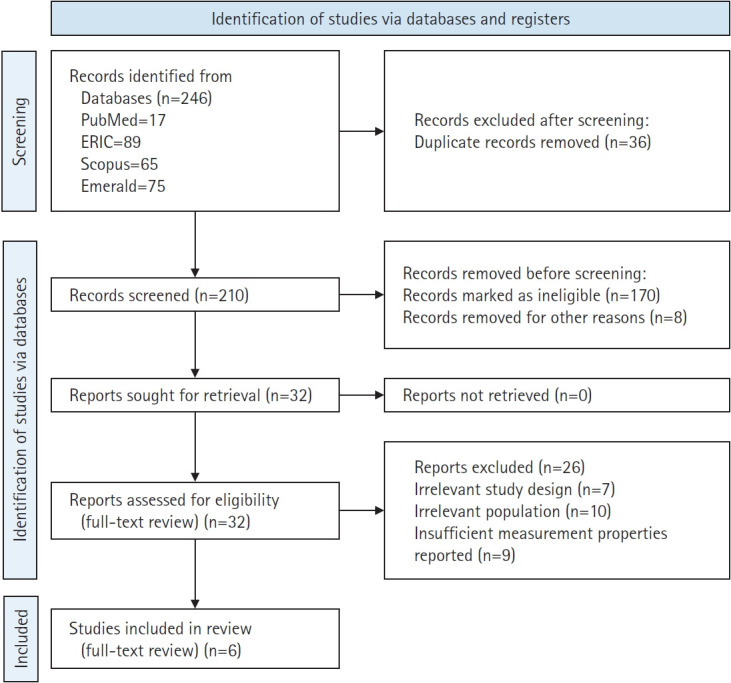
PRISMA (Preferred Reporting Items for Systematic Reviews and Meta-Analyses) flow diagram of the systematic review process. The diagram illustrates the stages of study selection, beginning with the identification of 246 records from 4 electronic databases (PubMed, ERIC, Scopus, and Emerald Insight). It details the screening process, the removal of 36 duplicate records, and the exclusion of 178 studies based on title and abstract screening. The final stage shows the 32 full-text articles assessed for eligibility, resulting in the inclusion of 6 studies in the qualitative synthesis based on predefined inclusion and exclusion criteria.

All identified records were imported into a reference management system for deduplication. Two reviewers independently screened titles and abstracts against the eligibility criteria. Any discrepancies or uncertainties regarding study inclusion were resolved through detailed discussion and mutual agreement between the 2 reviewers. Full-text articles were then independently assessed by the same 2 reviewers. Disagreements at the full-text stage were similarly resolved through discussion and consensus to ensure a robust final selection. No automation tools were used. The process is illustrated in Fig. 1.

### Data collection process

Data were extracted using a structured form. To ensure consistency and shared understanding, the reviewers conducted joint pilot extraction of 2 randomly selected studies and refined the form as necessary before proceeding to full extraction. Two reviewers independently collected data from each report. Extracted information was cross-checked for accuracy, and any discrepancies were resolved through discussion and consensus between the 2 reviewers. No automation tools were used.

### Data items

The primary outcomes were measurement properties defined by the COSMIN taxonomy [11]. Other variables included author(s), year, country, instrument name, target population, sample size, number of items, and domains assessed. Missing information was addressed by contacting authors when necessary or was noted as “not reported.”

### Study risk of bias assessment

Methodological quality was assessed using the COSMIN Risk of Bias checklist, 2018 update [11]. Two reviewers independently evaluated each measurement property. Each property was rated as very good, adequate, doubtful, or inadequate using the “worst score counts” principle. Discrepancies were resolved through consensus.

### Effect measures

As this was a systematic review of measurement instruments, the effect measures included psychometric indices such as Cronbach’s alpha for internal consistency, intraclass correlation coefficients for reliability, and structural validity indices, such as comparative fit index (CFI) and root mean square error of approximation (RMSEA).

### Synthesis methods

Measurement properties were assessed against COSMIN criteria for good measurement properties and rated as sufficient (+), insufficient (–), or indeterminate (?). A narrative synthesis approach was used to summarize findings because of heterogeneity in study designs and instruments. Meta-analysis was not conducted; therefore, assessment of statistical heterogeneity or sensitivity analysis was not applicable.

### Reporting bias assessment

Reporting bias was not formally assessed using funnel plots because of the limited number of studies for each instrument and the lack of sufficient quantitative data required for such testing, consistent with COSMIN guidelines.

### Certainty assessment

The overall quality of evidence for each measurement property was graded using the COSMIN-modified Grading of Recommendations Assessment, Development, and Evaluation (GRADE) approach [11]. Evidence was downgraded on the basis of risk of bias, inconsistency, imprecision, and indirectness and was classified as high, moderate, low, or very low quality.

## Results

### Study selection

The systematic search identified 246 records across PubMed, ERIC, Scopus, and Emerald Insight. After duplicates were removed, 210 records remained for title and abstract screening. Of these, 178 studies were excluded because they did not meet the eligibility criteria, primarily because they focused on student ethics rather than teaching ethics, used conceptual or theoretical designs, involved instruments developed for noneducational contexts, or lacked psychometric data. Thirty-two studies underwent full-text review. Of these, 26 studies were excluded for the following reasons: irrelevant study design (n=7), irrelevant population (n=10), and insufficient reporting of measurement properties (n=9). Finally, 6 studies met all inclusion criteria and were included in the final synthesis. The study selection process is illustrated in the PRISMA flow diagram (Fig. 1).

### Study characteristics

The 6 included studies were conducted in Iran, Türkiye, the United States and collectively involved more than 3,000 participants. The included instruments and their corresponding primary studies were the Ethical Sensitivity Scale Questionnaire (ESSQ) [12], Ethical Leadership Scale (ELS) [13], Ethical Evaluation Questionnaire (EEQ) [14], Teaching-Profession Ethical Principles Inventory (TEPI) [15], Teachers’ Compliance with Professional Ethics in Relations with Students Scale (TCPERSS) [16], and Moral Competency Inventory (MCI) [17]. Target populations consisted primarily of teachers and faculty members. The number of items ranged from 10 to 56, and the number of domains ranged from 1 to 7. Most instruments were self-report Likert-type questionnaires, except for the TCPERSS scale, which incorporates peer assessment. Detailed characteristics are presented in Table 4.

### Risk of bias in studies

Overall methodological quality, assessed using the COSMIN Risk of Bias checklist, ranged from adequate to very good across the 6 included studies (Table 5). Content validity and structural validity were generally supported by robust exploratory and confirmatory factor analyses. However, some subscales in the ESSQ [12] and TEPI [15] demonstrated Cronbach’s α values below 0.70. Formal indices of measurement error were rarely reported, although several studies addressed potential sources of error through item analysis. Direct comparisons with gold-standard instruments were limited, and criterion-related evidence was often inferred from theoretically consistent correlations. Although internal consistency and structural validity were robust across most tools, cross-cultural validity remained a significant limitation. Based on the COSMIN risk of bias assessment, instruments such as the MCI and ESSQ were downgraded to “adequate” because their validation was primarily conducted within single-country contexts, such as Türkiye and Iran. There was a notable absence of multigroup invariance testing across different linguistic and cultural groups.

### Results of individual studies

Internal consistency estimates across individual studies ranged from acceptable to excellent (Cronbach’s α =0.74–0.97). Structural validity was supported for the EEQ, TEPI, TCPERSS, and MCI, with acceptable to strong model fit indices. Reliability evidence primarily reflected internal consistency and split-half coefficients, as test–retest or inter-rater reliability was not consistently reported. Detailed psychometric findings for each instrument are summarized in Table 6.

### Results of syntheses

Across the included instruments, structural validity and internal consistency emerged as the most consistently supported properties. The content validity of the included instruments was synthesized based on the available reporting. Instruments such as the EEQ, TEPI, and TCPERSS showed sufficient evidence (+) through expert-led development. However, for the ESSQ, ELS, and MCI, the evidence was rated as indeterminate (?) because of a lack of explicit reporting on item comprehensiveness and pilot-testing feedback, as required by COSMIN standards. In contrast, construct validity (hypothesis testing) and temporal stability were rarely examined. Although the instruments demonstrated acceptable psychometric support within their respective validation contexts, none were specifically designed for health professions education. A comparative synthesis of measurement properties and overall quality of evidence is presented in Table 7.

### Reporting biases

Reporting bias was not formally assessed using funnel plots because of the limited number of studies included for each instrument and the lack of sufficient quantitative data required for such statistical testing, which is consistent with COSMIN guidelines for systematic reviews of outcome measures.

### Certainty of evidence

Using the COSMIN-modified GRADE approach, all 6 instruments were rated as having moderate-quality evidence (Table 8). Downgrading was primarily due to reliance on single-study evidence, limited cross-context validation, and the absence of temporal stability and formal measurement error assessment. Indirectness was rated as moderate because the instruments were validated within specific national or educational settings.

## Discussion

### Key results

Six instruments met the inclusion criteria from 246 records: the ESSQ, ELS, EEQ, TEPI, TCPERSS, and MCI. These studies were conducted in Iran, Türkiye, the United States and involved more than 3,000 participants. Internal consistency was acceptable to excellent (Cronbach’s α=0.74–0.97). Structural validity was generally supported. Reports on construct validity, test–retest reliability, and cross-cultural validation were inconsistent. As a result, all instruments received moderate GRADE evidence ratings. Notably, none were developed specifically for health professions education, despite their potential relevance to this field.

### Interpretation

This systematic review identified and critically appraised 6 instruments assessing the ethics of teaching. The findings indicate that although existing tools such as the TEPI, TCPERSS, EEQ, and MCI demonstrate generally acceptable structural validity and internal consistency, significant gaps remain. The absence of test–retest reliability and measurement error data limits understanding of measurement stability over time. Furthermore, the fragmentation between instruments focusing on “moral role modeling” and those focusing on “ethical teaching practices” suggests that no single tool yet captures the full multidimensional nature of the ethics required in complex educational and clinical environments.

### Comparison with previous studies

The generally supportive psychometric evidence found in this review aligns with broader trends in educational evaluation, in which structural validity and internal consistency are frequently prioritized. However, unlike previous reviews in general education, this study highlights a critical contextual gap: none of the identified instruments were originally developed for health professions education. This is consistent with findings in the medical education literature suggesting that general pedagogical tools often fail to capture unique clinical ethical dimensions, such as patient safety and professional power dynamics [8,11].

Synthesis of the 6 identified instruments revealed distinct differences in their focal contexts and ethical dimensions. Instruments such as the ESSQ primarily target internal moral perception, which remains highly abstract. Meanwhile, the ELS and EEQ emphasize leadership roles within a general organizational framework, namely workplace ethics, rather than direct instructional interactions. Furthermore, although the TEPI and MCI assess attitudes and competencies, they have significant limitations when applied to the health professions context, which is substantially more complex than general education.

The heightened complexity of health professions education stems from its workplace-based learning context, which is often characterized by high-pressure environments. Educators must simultaneously fulfill dual roles as instructors and professional supervisors. Consequently, the teacher–student relationship extends beyond the classroom into critical decision-making situations that directly affect life and safety. Such contexts may exacerbate the power imbalance more severely than general education settings; thus, a lack of teaching ethics can directly jeopardize student safety and well-being.

Accordingly, this study suggests that the Teachers’ Compliance with Professional Ethics in Relations with Students Scale (TCPERSS) appears to be one of the most promising instruments currently available for the health professions education context [16]. It is a unique tool focused specifically on professional ethics compliance within the teacher–student relationship. Crucially, it incorporates a Student Form, which may provide a more objective reflection of an educator’s role modeling under these complex conditions than self-assessment alone.

When evaluated against universal ethical principles, most instruments, including the TCPERSS, effectively reflect justice and beneficence. However, significant gaps remain regarding nonmaleficence and student autonomy. Specifically, with regard to nonmaleficence, existing tools do not adequately cover the protection of students from harassment or pressure to perform inappropriate actions during classroom instruction or, more importantly, during clinical practice, which is the most vulnerable setting in health professions education. In addition, there is a lack of measurement of student autonomy, particularly students’ participation in decision-making and their freedom to express dissenting opinions in urgent clinical contexts. Therefore, although the TCPERSS currently serves as the most robust foundation, future instrument development should incorporate items focused on protecting student rights and safety within actual practice environments to fully align the ethics of teaching with universal international standards. The lack of cross-cultural evidence is particularly critical in health professions education. Ethical perceptions in teaching often vary between Western-centric models, which emphasize individual autonomy, and Eastern-oriented contexts, which may prioritize hierarchy and collective responsibility. Future research should prioritize transcultural validation to ensure that these instruments are sensitive to the power dynamics inherent in diverse global educational settings.

### Limitations

This review was restricted to studies published in English, potentially excluding relevant instruments developed and validated in other linguistic contexts. In addition, the conclusions are constrained by incomplete reporting of reliability over time and measurement error in the original studies, which hindered a full COSMIN-based meta-synthesis.

Another limitation is that most identified instruments rely on self-report measures, which are inherently susceptible to social desirability bias and may lead educators to overreport ethical behaviors. However, it is noteworthy that the TCPERSS scale uses multisource assessment, which may provide a more objective evaluation of ethical compliance and mitigate some of the biases associated with self-reporting. Future research should continue to promote such multisource assessments, including student and peer evaluations, to ensure a more comprehensive and accurate reflection of ethical conduct in health professions education.

### Implications

For educational practice, existing instruments offer a starting point but require further validation before broader implementation. For future research, there is a pressing need to develop or adapt a context-specific instrument for health professions education that integrates both pedagogical ethics and clinical professional responsibilities. Such an instrument should undergo rigorous evaluation using the COSMIN framework to ensure that it is psychometrically sound for diverse healthcare settings.

## Conclusion

The ethics of teaching is a critical multidimensional construct that shapes educational quality and professional integrity. This review synthesized evidence on 6 instruments—the ESSQ, ELS, EEQ, TEPI, TCPERSS, and MCI—and found adequate psychometric support while also highlighting areas for further research, particularly construct validity (hypothesis testing) and cross-cultural validation. Notably, the lack of instruments specific to health professions education represents a significant opportunity for future development. Strengthening measurement in this area is essential for promoting evidence-based educational evaluation and advancing ethical teaching practices in both academic and clinical healthcare settings.

## Figures and Tables

**Figure f2-jeehp-23-12:**
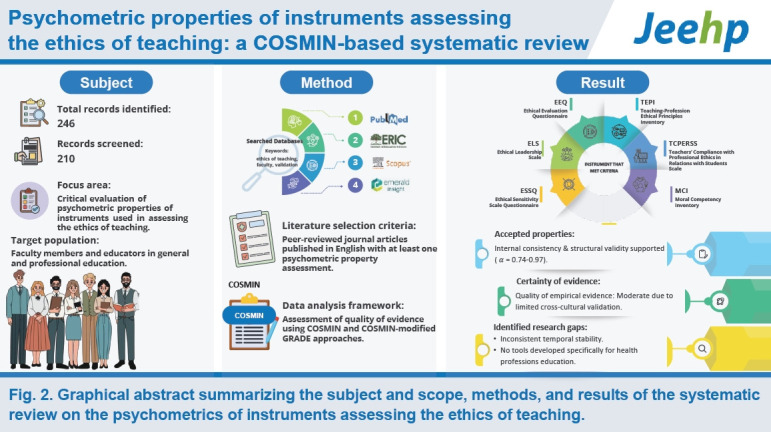


**Table 1.** Inclusion and exclusion criteria for study selection

Category	Inclusion criteria	Exclusion criteria
Population	Faculty members, instructors, or educators in health professions and general education	Instruments focusing primarily on the ethics of students or patients
Instrument	Instruments measuring the ethics of teaching or instructors’ ethical behavior	Instruments unrelated to teaching
Measurement properties	Reported reliability, validity, test–retest reliability, or cross-cultural consistency	Conceptual papers or studies without statistical data
Language	English	Other languages that could not be translated

Only peer-reviewed journal articles were included to ensure the quality of psychometric evidence.

**Table 2.** Search concepts and keywords used in the systematic review

Concept	Keywords	MeSH terms
1. Construct of interest	Ethics of teaching; ethical teaching behavior; teaching ethics; moral role model in teaching; ethical decision-making	Ethics, Professional; Morals
2. Target population	Faculty; educators; nursing educators; health professions educators; medical education	Faculty; Faculty, Nursing; Health Occupations/education
3. Measurement properties	Validity; reliability; psychometric properties; construct validity; internal consistency; test–retest reliability	Reproducibility of Results; Psychometrics
4. Methodology	Questionnaire; tool; assessment; measure; instrument; scale	Surveys and Questionnaires

MeSH, Medical Subject Headings.

**Table 3.** Search syntax and results by database

Database	Key concept	Search syntax	Records found
PubMed	#1 Construct of Interest	("Ethics of teaching"[Title/Abstract] OR "ethical teaching behavior"[Title/Abstract] OR "teaching ethics"[Title/Abstract]	624
		OR "ethical sensitivity"[Title/Abstract] OR "ethical leadership"[Title/Abstract] OR "moral intelligence" [Title/Abstract] OR "ethical awareness"[Title/Abstract] OR "teaching evaluation"[Title/Abstract])	
	#2 Target Population	("Faculty"[Title/Abstract] OR "educators"[Title/Abstract] OR "nursing educators"[Title/Abstract] OR "health professions educators"[Title/Abstract]	65,031
		OR "nursing students"[Title/Abstract] OR "teachers" [Title/Abstract])	
	#3 Measurement Properties	("validity"[Title/Abstract] OR "reliability"[Title/Abstract] OR "psychometric properties"[Title/Abstract])	187,439
	#4 Methodology	("questionnaire"[Title/Abstract] OR "instrument" [Title/Abstract] OR "scale"[Title/Abstract])	855,765
	Combined	(#1 AND #2 AND #3 AND #4)	17
ERIC	#1-#4 Combined	(#1 AND #2 AND #3 AND #4) AND ("Peer reviewed only AND Full text available on ERIC")	89
Scopus	TITLE-ABS-KEY("ethics of teaching" OR "ethical teaching behavior" OR "teaching ethics") AND TITLE-ABS-KEY("faculty" OR "educators" OR "nursing educators") AND TITLE-ABS-KEY("validity" OR "reliability" OR "psychometric properties") AND TITLE-ABS-KEY("questionnaire" OR "tool" OR "assessment" OR "instrument" OR "scale")		65
Emerald	(TS=("ethical teaching" OR "teaching ethics" OR "ethical leadership" OR "ethical awareness" OR "moral intelligence"))		75
	AND		
	(TS=("faculty" OR "educators" OR "teachers" OR "university" OR "higher education"))		
	AND		
	(TS=("scale" OR "questionnaire" OR "instrument" OR "inventory" OR "survey"))		

#1–4, search concept sequences; TITLE-ABS-KEY, search within title, abstract, and keywords; TS, topic search.

**Table 4.** Characteristics of the instruments and studies included in the review

Authors (year), country	Name of instrument	Population/language	Sample size (no. of participants)	Items	Domains
Gholami & Tirri (2012), Iran	ESSQ	Teachers/Persian	556	28	7 domains: emotional expression, caring, perspective-taking, group differences, bias prevention, interpretation, and consequences
Ariail et al. (2023), USA	ELS	University faculty/English	539	10	1 domain: ethical leadership
Toker Gokce (2017), Türkiye	EEQ	Teachers/Turkish	346	35	7 domains: religious, personal life, social life, moral, Machiavellianism/utilitarianism, intrinsic, and extrinsic ethics
Koçyiğit & Karadağ (2017), Türkiye	TEPI	Teachers/Turkish	305	56	6 domains: student ethics, sensitivity, in-school behavior, discrimination, controversial issues, and stakeholders
Arslan et al. (2025), Türkiye	TCPERSS	Teachers and students/Turkish	1,760^a)^	18	1 domain: ethical principles in teacher–student relations (8 subdimensions)
Toprak & Karakus (2018), Türkiye	MCI	Primary teachers/Turkish	773	23	4 domains: integrity, responsibility, compassion, and forgiveness

ESSQ, Ethical Sensitivity Scale Questionnaire; ELS, Ethical Leadership Scale; EEQ, Ethical Evaluation Questionnaire; TEPI, Teaching-Profession Ethical Principles Inventory; TCPERSS, Teachers’ Compliance with Professional Ethics in Relations with Students Scale; MCI, Moral Competency Inventory.

a) Total sample size across teacher and student forms in both stages.

**Table 5.** Methodological quality of studies using the COSMIN risk of bias checklist

Domain/instrument	ESSQ	ELS	EEQ	TEPI	TCPERSS	MCI
Content validity	Adequate	Adequate	Very good	Very good	Very good	Very good
Structural validity	Adequate	Adequate	Very good	Very good	Very good	Very good
Internal consistency	Adequate	Very good	Very good	Very good	Very good	Very good
Cross-cultural validity	Adequate	Unknown (?)	Unknown (?)	Unknown (?)	Adequate	Adequate
Reliability	Adequate	Adequate	Very good	Very good	Very good	Very good
Measurement error	Adequate	Doubtful	Adequate	Very good	Very good	Adequate
Construct validity (hypothesis testing)	Doubtful	Very good	Adequate	Doubtful	Doubtful	Adequate
Responsiveness	Inadequate	Inadequate	Inadequate	Inadequate	Inadequate	Inadequate

COSMIN, consensus-based standards for the selection of health measurement instruments; ESSQ, Ethical Sensitivity Scale Questionnaire; ELS, Ethical Leadership Scale; EEQ, Ethical Evaluation Questionnaire; TEPI, Teaching-Profession Ethical Principles Inventory; TCPERSS, Teachers’ Compliance with Professional Ethics in Relations with Students Scale; MCI, Moral Competency Inventory.

**Table 6.** Summary of validity and reliability evidence for the included instruments

Name of instrument	Content validity	Construct validity	Construct validity (hypothesis testing)	Structural validity	Internal reliability	Test–retest	Inter-rater
1. ESSQ	?	+	?	+	?	NA	NA
(Gholami & Tirri, 2012)	Indeterminate	CFA 7-factor fit	No gold standard	CFI 0.88, RMSEA 0.06	α=0.84 (sub: 0.45–0.69)		
2. ELS	?	+	?	?	+	NA	NA
(Ariail et al., 2023)	Indeterminate	r=0.61 (P<0.01)	No benchmark	Not revalidated	α=0.95 (excellent)		
3. EEQ	+	+	?	+	+	NA	NA
(Toker Gokce, 2017)	Sufficient	EFA/CFA 7-domain	Inter-domain r only	KMO 0.88, Var 58%–61%	α=0.74–0.90		
4. TEPI	+	+	?	+	+/–	NA	NA
(Koçyiğit & Karadağ, 2017)	Sufficient	MoNE framework fit	No comparison scale	EFA KMO 0.91	α=total 0.95		
5. TCPERSS	+	+	?	+	+	NA	NA
(Arslan et al., 2025)	Sufficient	One-factor MoNE model	Theoretical linkage	CFI 0.94, RMSEA 0.07	α=0.94–0.97		
6. MCI	?	+	?	+	+	NA	NA
(Toprak & Karakus, 2018)	Indeterminate	EFA/CFA 4-factor	Subscale r=0.67–0.86	RMSEA 0.04, CFI 0.91	α=total 0.88		

?, indeterminate; +, sufficient; –, insufficient; NA, not applicable/not reported; CFA, confirmatory factor analysis; CFI, comparative fit index; RMSEA, root mean square error of approximation; α, Cronbach’s alpha; r, Pearson correlation coefficient; EFA, exploratory factor analysis; KMO, Kaiser-Meyer-Olkin; Var, variance explained; MoNE, Ministry of National Education.

**Table 7.** Overall justification and quality of evidence for the included instruments

Instrument	Overall rating^a)^	QoE^b)^	Justification
ESSQ	+	Moderate	Acceptable structural validity and internal consistency; however, some subscales fell below α=0.70 and evidence derived from a single cultural context.
ELS	+	Moderate	Strong internal consistency and theory-supported correlations; however, structural validity was not re-examined and temporal stability was not assessed.
EEQ	+	Moderate	Robust factorial validation (EFA and CFA) and good reliability; limited by single-country evidence and absence of test–retest data.
TEPI	+	Moderate	Multi-phase development and strong factor structure; some subscales below threshold and lack of longitudinal reliability evidence.
TCPERSS	+	Moderate	Excellent internal consistency and dual-form alignment; no formal measurement error or temporal stability assessment.
MCI	+	Moderate	Supported factor structure and adequate reliability in adaptation study; limited cross-context validation and no stability testing.

QoE, quality of evidence; ESSQ, Ethical Sensitivity Scale Questionnaire; α, Cronbach’s alpha; ELS, Ethical Leadership Scale; EEQ, Ethical Evaluation Questionnaire; EFA, exploratory factor analysis; CFA, confirmatory factor analysis; TEPI, Teaching-Profession Ethical Principles Inventory; TCPERSS, Teachers’ Compliance with Professional Ethics in Relations with Students Scale; MCI, Moral Competency Inventory.

a) Overall rating: “+” indicates acceptable psychometric support within the reviewed context; “–” indicates insufficient support

b) QoE was determined using modified GRADE principles considering risk of bias, consistency, imprecision, and indirectness.

**Table 8.** Summary of evidence and quality grading based on the GRADE approach

Instrument	Risk of bias	Consistency	Imprecision	Indirectness	Overall GRADE
ESSQ	Moderate	Moderate	Moderate	Moderate	Moderate
ELS	Moderate	High	Moderate	Moderate	Moderate
EEQ	Moderate	High	Moderate	Moderate	Moderate
TEPI	Moderate	High	Moderate	Moderate	Moderate
TCPERSS	Moderate	High	Low–Moderate	Moderate	Moderate
MCI	Moderate	High	Moderate	Moderate	Moderate

GRADE levels: high (very confident that the true effect lies close to that of the estimate); moderate (moderately confident in the effect estimate); low (confidence in the effect estimate is limited); and very low (very little confidence in the effect estimate). GRADE, Grading of Recommendations Assessment, Development and Evaluation; ESSQ, Ethical Sensitivity Scale Questionnaire; ELS, Ethical Leadership Scale; EEQ, Ethical Evaluation Questionnaire; TEPI, Teaching-Profession Ethical Principles Inventory; TCPERSS, Teachers’ Compliance with Professional Ethics in Relations with Students Scale; MCI, Moral Competency Inventory.

## Supplementary materials

Supplement 1. Search strategy, selection criteria, COSMIN risk of bias, study characteristics, psychometric results, GRADE and summary of this study.

Supplement 2. Audio recording of the abstract.
